# Novel *SIM1* Variants Expanding the Spectrum of SIM1-Related Obesity

**DOI:** 10.3390/ijms27010533

**Published:** 2026-01-05

**Authors:** Idris Mohammed, Wesam S. Ahmed, Tara Al-Barazenji, Hajar Dauleh, Donald R. Love, Khalid Hussain

**Affiliations:** 1 College of Health & Life Sciences, Hamad Bin Khalifa University, Doha P.O. Box 34110, Qatar; imohammed-c@sidra.org (I.M.); wisamyejo@yahoo.com (W.S.A.); 2 Division of Endocrinology, Department of Pediatric Medicine, Sidra Medicine, Doha P.O. Box 26999, Qatar; talbarazenji@sidra.org (T.A.-B.); hdauleh1@sidra.org (H.D.); 3 Division of Genetic Pathology, Department of Pathology, Sidra Medicine, Doha P.O. Box 26999, Qatar; dlove@sidra.org

**Keywords:** *SIM1*, monogenic obesity, leptin-melanocortin, hyperphagia, early-onset obesity, Prader-Willi-like syndrome

## Abstract

Monogenic forms of severe early-onset obesity often involve genetic disruptions in the hypothalamic leptin-melanocortin pathway. Pathogenic variants in the *SIM1* gene, a key transcription factor required for the development of the paraventricular nucleus, are a known cause of Prader–Willi-like syndrome, characterized by hyperphagia, severe obesity, and developmental delay. We performed targeted next-generation sequencing of 52 obesity-associated genes on a cohort of pediatric patients with severe early-onset obesity. Identified variants were analyzed for population frequency and predicted pathogenicity using in silico tools. The structural impact of the novel missense variants was assessed using protein domain modeling with AlphaFold3. We identified five rare *SIM1* variants in eleven patients. Four were heterozygous nonsynonymous variants: one frameshift in the bHLH domain (p.Ser18Ter), one frameshift in the Per-ARNT-Sim domain (p.His143Ter), and two missense variants, p.Pro30Ala and p.Ser663Leu. Structural modeling suggested that the missense variants are likely to disrupt critical protein–protein interactions. The fifth variant was a synonymous change, c.1173G>A, p.(Ser391Ser), which was detected in five unrelated patients. Bioinformatic analysis predicted that this variant could alter splicing. Structural modeling suggested that the missense variants interfere with SIM1 function. This study expands the mutational spectrum of SIM1-linked monogenic obesity, reporting novel likely pathogenic frameshift variants, a missense variant, and a recurrent synonymous variant with a potential splice-site effect. The majority of the variants are predicted to affect the SIM1 protein. Our findings strengthen the critical role of the *SIM1* gene in hypothalamic development and energy homeostasis. The results underscore the importance of including the *SIM1* gene in genetic testing panels for children with severe obesity and hyperphagia, enabling precise diagnosis and potential future personalized management. Functional in vitro or in vivo validation of these variants is required to confirm their pathogenicity.

## 1. Introduction

Obesity is a worldwide epidemic with rates nearly tripled over the last three decades among adults and increased almost five times among children and adolescents [1]. Although the primary cause of obesity is complex, an interplay between environmental and polygenic factors, there is considerable evidence of a monogenic contribution [2]. The vast majority of monogenic forms of obesity are due to genetic defects in the hypothalamic leptin-melanocortin signalling pathway, a pathway crucial in regulating appetite, energy regulation, and body weight [3]. Pathogenic variants in monogenic obesity disrupt a single gene and lead to severe early-onset obesity, accompanied by hyperphagia and some endocrine disorders [4].

One of the monogenic obesity genes is the single-minded homolog one gene (*SIM1*), which encodes a transcription factor located on the long arm of chromosome 6 (6q16.3) and required for neurogenesis, particularly in the development and function of the paraventricular nucleus (PVN) [5]. The paraventricular nucleus is a crucial component of the central nervous system (CNS), playing a vital role in maintaining energy balance [6]. Loss-of-function and haploinsufficiency of the *SIM1* gene lead to a Prader–Willi-like phenotype (PWL). Patients with PWL syndrome carry deletions in the long arm of chromosome 6 (6q16), which contains approximately ten genes, including the *SIM1* gene [7,8]. Recently, several studies have linked the PWL syndrome specifically to SIM1 haploinsufficiency. Some of the common phenotypes associated with PWL syndrome include severe early-onset obesity, hyperphagia, and developmental delay [5,9]. A study by Yang et al. demonstrated that mice with haploinsufficiency of SIM1 exhibited abnormal hypothalamic development, whereas overexpression of Sim1 led to reduced food intake [10]. Mice homozygous for Sim1 deficiency die prenatally, whereas heterozygosity and haploinsufficiency lead to the development of early-onset obesity, hyperphagia, hyperinsulinemia, and increased linear growth [7]. Haploinsufficiency and loss-of-function mutations in *SIM1* in humans have also been shown to lead to severe early-onset obesity, hyperphagia, and developmental delay [8,11,12].

SIM1 heterodimerizes with aryl hydrocarbon receptor nuclear translocator 2 (ARNT2), which is essential for SIM1 to function in the development of the paraventricular nucleus [13]. The SIM1-ARNT2 heterodimer is crucial for proper hypothalamic development, particularly in regulating neuroendocrine and metabolic functions [14]. When either SIM1or ARNT2 is absent, the neuronal precursors develop normally, but they cannot assemble into the proper neuroendocrine structures, specifically the paraventricular and supraoptic nucleus (SON) in the anterior hypothalamus. This leads to failed neuronal organisation despite normal precursor development [15]. Hence, loss of function or haploinsufficiency of the SIM1 protein results in a reduction in the MC4R neuronal populations in the paraventricular nuclei, contributing to hyperphagia and obesity, similar to the MC4R deficiency [7,16].

To date, approximately 30 patients have been reported to have *SIM1* variants associated with severe obesity (Table 1). In this study, we describe eleven patients with early-onset obesity who underwent genetic testing and were found to carry both nonsynonymous and synonymous variants in the *SIM1* gene.

## 2. Methodology

This study included 246 probands with severe early-onset obesity (BMI percentile > 95th). One hundred thirty-nine probands were males (57%), and 107 (43%) were females. The inclusion criteria were an age at obesity onset before 10 years old. Two-thirds of the probands (*n* = 163, 68%) were under five years old, and the mean age of the probands was 4.3 years. The institutional research board (IRB) approved the study (IRB reference number 1702007592, approval date: 2 June 2023), and signed assent, consent forms, and parental permission were obtained. Peripheral blood was collected, and genomic DNA was extracted following the manufacturer’s protocol (#51104, Qiagen, Hilden, Germany). Genomic DNA concentrations and purities were assessed using a Nanodrop 2000 spectrophotometer (Thermo Scientific, Waltham, MA, USA). For next-generation sequencing, exonic regions of all genes of interest were captured using an optimised set of DNA hybridisation probes. The captured DNA was sequenced on the Illumina NovaSeq 6000 platform (Illumina, San Diego, CA, USA). We targeted regions, including all exons and flanking regions, of 52 genes associated with obesity—genes regulating energy balance in the leptin-melanocortin pathway, genes involved in the differentiation and proliferation of hypothalamic nuclei, genes linked to monogenic ciliopathy diseases, and genes essential for adipocyte differentiation. The sequencing of these cases was part of a cohort that underwent panel sequencing, specifically a comprehensive monogenic obesity panel by a company (Prevention Genetics, Marshfield, WI, USA). According to the company’s report, the average NGS coverage was 538× and the base coverage fraction was 100.0%. The minimum NGS coverage was ≥20× for all exons and ±10 bp in the flanking regions. A summary of all 52 genes included in the targeted gene panel, along with the analysis method, is provided in our previously published research [24].

To assess the structural impact of pathogenic *SIM1* missense variants, the protein sequences of SIM1 (UniProt entry: P81133) and ARNT2 (UniProt entry: Q9HBZ2) were obtained [25], and the structures of their bHLH, PAS, and PAC domains were predicted using AlphaFold3 [26]. For SIM1, residues 1–350 were modeled; for ARNT2, residues 60–455 were modeled. Predictions were performed in both monomeric and heterodimeric states using the same seed value for all runs. The resulting structures were visualized and analyzed in PyMOL (Molecular Graphics System, Version 2.0.0, Schrödinger, LLC, New York, NY, USA; https://www.pymol.org).

## 3. Results

In this study, we identified five rare variants in the *SIM1* gene (NM_005068.3) among severely obese children, most of whom were under the age of five years. Four of these variants were nonsynonymous: c. 52_53delAG, p.(Ser18Ter) (Chr6:100,911,293-100,911,294delTC), c.88C>G, p.(Pro30Ala) (Chr6:100911257G/C), c.1988C>T, p.(Ser663Leu) (Chr6:100838550G/A), and c.428_434del, p.(His143Ter) (Chr6.100897498-1008974504delTAGTTGG), each found in unrelated patients. At the same time, we identified one synonymous variant, c.1173G>A, p.(Ser391Ser) (Chr6:100841760-G/A), that was detected in five patients. All the above-mentioned variants are based on GRCh37/hg19 genomic coordinates.

**CASES**
*Case1: c. 52_53delAG, p.(Ser18Ter)*

A 13-year-old boy weighing 112.8 kg (BMI 35.7 kg/m^2^, z-score of 3.31), placing him in the 99.5th BMI percentile for his age and sex. The patient presented with severe obesity characterised by both peripheral and central adiposity and had prediabetes. His liver ultrasound revealed nonalcoholic steatohepatitis, persistent hepatomegaly, and diffuse fatty infiltration of the liver. He exhibited elevated liver enzymes (ALT, AST, and GGT). The patient also showed acanthosis nigricans on the neck and beneath the arms. Genetic testing identified a novel frameshift deletion in the *SIM1* gene, c. 52_53del, p.(Ser18Ter). This variant is located within a highly conserved domain, the basic helix-loop-helix (bHLH) domain of SIM1, which plays a crucial role in hypothalamic development, particularly in the paraventricular nucleus, which regulates appetite and metabolism.

*Case 2: c.88c>g, p.(Pro30Ala)*

A case involving a 15-year-old boy with a birth weight of 3.3 kg who started gaining weight at age 5. His current weight is 159 kg (BMI 47.3 kg/m^2^, Z-score 4.11), placing him at the 100th percentile for his age and sex. Liver ultrasound revealed increased echogenicity, indicating diffuse fatty infiltration of the liver. The patient has acanthosis nigricans on his neck. Laboratory tests showed elevated ALT levels. His genetic analysis identified a novel heterozygous nonsynonymous variant, c.88c>g, p.(Pro30Ala), in the *SIM1* gene, located within the conserved basic helix-loop-helix (bHLH) domain (Table 2A). This variant is predicted to be deleterious by several in silico tools, SIFT (https://sift.bii.a-star.edu.sg/ (accessed on 28 September 2025)), PolyPhen2 (http://genetics.bwh.harvard.edu/pph2/ (accessed on 28 September 2025)), and MutationTaster (https://www.mutationtaster.org/), and was not found in the gnomAD (https://gnomad.broadinstitute.org/) or TOPMed population databases (https://bravo.sph.umich.edu/).

*Case 3. c.428_434del, p.(His143Ter18)*

A 13-year-old child who started gaining weight at age two currently weighs 108.6 kg with a BMI of 48 kg/m^2^. The patient presented with severe obesity, hepatomegaly, hepatic steatosis, pre-diabetes, and dysmorphic features such as hydrocephalus, an elongated face, hypertelorism, and brachydactyly. Signs of insulin resistance and obstructive sleep apnea (OSA) were observed, along with severe acanthosis nigricans on the back of the neck. Genetic analysis revealed a pathogenic novel frameshift deletion, c.428_434del, p.(His143Ter18). The deletion of seven nucleotides occurs within a highly conserved domain among species, the PER-ARNT-SIM (PAS) domain, resulting in a truncated protein. This results in the loss of the PAS and transactivation motifs of the SIM1 protein.

*Cases 4, 5 and 6 c.1988C>T, p.(Ser663Leu)*

We detected a heterozygous missense variant, c.1988C>T, p.(Ser663Leu), in the *SIM1* gene. The variant has a minor allele frequency (MAF) of 0.000003977 in gnomAD. In silico prediction tools predicted conflicting pathogenicity interpretations, with SIFT and MutationTaster predicting it is deleterious, whereas PolyPhen2 predicts it is likely benign. The variant lies within a conserved domain of the SIM1 transactivation domain (Table 2B).

*Case 4:*

This 17-year-old adolescent was born at term with a birth weight of 3.5 kg and started gaining weight around the age of 10 years. Currently, the adolescent weighs 99.6 kg (BMI of 35.4 kg/m^2^). The patient presented with mild acanthosis nigricans around the neck and elevated ALT levels. The patient has a strong family history of obesity, with the father, older brother, and sister being obese.

*Case 5:*

A 5-year-old boy born at term with a birth weight of 3.5 kg. He started gaining weight in infancy. His current weight is 40.75 kg (BMI of 28.7 kg/m^2^, corresponding to a z-score SDS of 3.48 at the 99th percentile). The patient had marked hyperphagia and vomited after overeating. He presented with prediabetes (HbA1c of 5.8%) and elevated insulin and C-peptide levels, 398 pmol/L and 1475 pmol/L, respectively. He has a strong family history of obesity; his 12-year-old sister weighs 90 kg.

*Case 6:*

A 15-year-old adolescent began a significant weight gain trajectory around age 7, with a marked acceleration over the past year, resulting in a 30 kg weight gain and a current weight of 102 kg (BMI 32.35 kg/m^2^, Z-score + 2.67). The patient was diagnosed with type 2 diabetes at age 13 (HbA1c 7.3%), which has since progressed to poor glycemic control, with a current HbA1c of 9.6%. Genetic testing identified a heterozygous *SIM1* variant, c.988C>T (p.Ser663Leu), which is likely contributory to his severe, early-onset obesity and metabolic complications.

*Cases 7, 8, 9, 10, 11. (c.1173G>A, p.Ser391Ser)*

We detected a synonymous variant, c.1173G>A, p.(Ser391Ser) (6-100841760-C-T (GRCh37)) in five patients with early-onset obesity. The variant was identified in four unrelated families. Alamut Visual v2.11 predicts that the variant activates a cryptic acceptor site six base pairs downstream of the native acceptor site. The variant is rare, with MAF: 1/232,296 (0.000004305) in gnomAD.

*Case 7:*

An 11-year-old girl presented with childhood obesity, having begun gaining weight at age one. Her current weight is 71.7 kg (BMI: 32 kg/m^2^). The patient has obesity, hypothyroidism, and vitamin D deficiency. She has been taking Levothyroxine 25 mcg daily since age three for hypothyroidism and cholecalciferol 600 IU for vitamin D deficiency. Her mother is obese and diabetic, having developed gestational diabetes during her pregnancy.

*Case 8:*

A 10-year-old girl presented with childhood obesity and hyperphagia. She began gaining weight in infancy, and her current weight is 65 kg (BMI: 31.5 kg/m^2^).

*Case 9:*

A 15-year-old girl presented with severe obesity (136 kg, BMI: 48.8 kg/m^2^, and SDS: 3.2) and type 2 diabetes (HbA1c: 8.3). She had normal growth and development, with rapid weight gain beginning at age 10. Following this rapid weight gain, she developed breathing abnormalities and severe OSA. The patient experienced alveolar hypoventilation during sleep and showed evidence of hypothalamic dysfunction, including corticotropin deficiency. She had a strong family history of obesity, with a sister who developed early-onset obesity and a mother who underwent sleeve surgery for obesity.

*Case 10:*

A 17-year-old girl with severe obesity and insulin resistance. This patient is the sister of Case 8, mentioned above. Her weight is 103 kg, with a BMI of 37 kg/m^2^ and a BMI SDS of 2.85. She began gaining weight around age five. Her abdominal ultrasound showed a mild, diffuse increase in liver echogenicity, consistent with mild fatty infiltration. The pancreas appeared echogenic but otherwise normal, likely due to pancreatic lipomatosis (fatty infiltration). Her liver function tests revealed elevated ALT and AST levels at 33 IU/L and 34 IU/L, respectively.

*Case 11:*

A 14-year-old girl presented with early-onset obesity, subclinical hypothyroidism, snoring, acanthosis nigricans, and prediabetes (HbA1c 5.9%). The patient began gaining weight before age five. Her current weight is 106 kg (BMI 35 kg/m^2^). An abdominal ultrasound showed a mildly enlarged liver with hepatic steatosis. She has a strong family history of obesity, with her mother and three paternal uncles undergoing bariatric surgery for severe obesity. The patient’s demographics, genetic variants, in silico predictions, and ACMG classifications for all cases identified in this cohort are summarized in Table 3.

### Structural Predictions and Domain Organization of the Missense Variants

**Figure 1:**
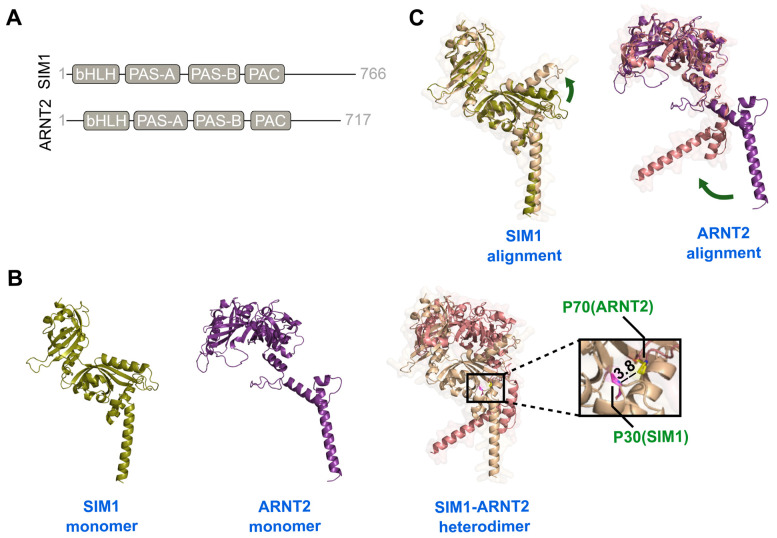
Structural prediction of SIM1 and its interaction with ARNT2. (**A**) Schematic representation of domain organization of full-length SIM1 and ARNT2, showing the bHLH, PAS-A, PAS-B, and PAC regions. (**B**) Cartoon representations of the predicted structures of SIM1 (residues 1–350) and ARNT2 (residues 60–455) in the monomeric state (**left and middle panels**, respectively) and as a heterodimer (**right panel**). The zoom-in inset shows the distance (in Å) between residue P30 of SIM1 (magenta sticks) and the bHLH/PAS-A loop of ARNT2, specifically residue Pro70 (yellow sticks). (**C**) Structural alignment of monomeric and heterodimeric states of SIM1 (left) and ARNT2 (right). Green arrows indicate conformational changes upon dimerization. Color scheme: SIM1 monomer, deep-olive; SIM1 heterodimer, wheat; ARNT2 monomer, violet-purple; ARNT2 heterodimer, salmon.

AlphaFold 3 modelling of SIM1 (residues 1–350) and ARNT2 (residues 60–455) reveals that both proteins possess the canonical bHLH–PAS architecture, comprising bHLH, PAS-A, PAS-B, and PAC domains. The N-terminal region of ARNT2 (residues 1–59) and the C-terminal segments of both proteins are predicted to be intrinsically disordered (Figure 1A). Structural predictions were generated for both monomeric and heterodimeric states of SIM1 and ARNT2. The resulting SIM1–ARNT2 heterodimer model exhibits extensive interdomain contacts across the bHLH and PAS regions. Notably, SIM1 residue P30 is located within a loop of the bHLH domain at the dimer interface, approximately 3.8 Å from the bHLH/PAS-A loop of ARNT2 (Figure 1B). This proximity suggests that P30 may contribute to the amino acid substitution and could alter the locus responsible for heterodimer formation. The substitution with alanine (P30A) could weaken this interface, impairing dimer formation. Conversely, residue Ser663 of SIM1 resides within the intrinsically disordered C-terminal region, precluding reliable structural inference. Nonetheless, since transcriptional regulation occurs through this disordered tail, the Ser663Leu variant might influence transcriptional activity rather than dimer formation.

Furthermore, comparison of monomeric and dimeric models showed conformational changes in both proteins that appear to facilitate dimerization. In SIM1, this involved movement of the first α-helix of PAS-A, while in ARNT2, a shift in the positioning of the bHLH domain was observed (Figure 1C). This indicates that direct dimer predictions, which we applied here, may more closely capture physiologically relevant conformations than docking approaches based solely on monomer structures.

## 4. Discussion

In this study, we identified five rare variants in the *SIM1* gene among eleven pediatric patients with severe early-onset obesity. Four were nonsynonymous variants: p.(Ser18Ter), p.(Pro30Ala), p.(Ser663Leu), and p.(His143Ter). One was a synonymous variant, c.1173G>A, p.(Ser391Ser), detected in five patients. These findings reinforce the critical role of *SIM1* in hypothalamic regulation of appetite and energy homeostasis, further supporting its involvement in monogenic obesity.

The *SIM1* gene encodes a transcription factor essential for the development of the PVN of the hypothalamus, a key region in regulating satiety and energy expenditure [7,15]. Our study identified frameshift deletions, p.(Ser18Ter) and p.(His143Ter), that likely cause haploinsufficiency, consistent with previous reports linking *SIM1* loss-of-function mutations to severe obesity and hyperphagia [8,9,11]. Notably, p.(His143Ter) truncates the PAS domain, which is crucial for protein–protein interactions and transcriptional activity [15]. Similarly, p.(Ser18Ter) is predicted to produce a null allele due to a premature termination codon, which is expected to cause a complete loss of the protein either through nonsense-mediated decay (NMD) or the generation of a non-functional, severely truncated protein, thereby abolishing its function. A non-functional SIM1 protein impairs DNA binding and dimerization with ARNT2, a partner necessary for proper hypothalamic development [13,15]. These findings are consistent with those from murine models, where Sim1 haploinsufficiency causes hyperphagia, obesity, and a reduction in PVN neuronal populations [7,16].

Additionally, we report a missense variant, p.(Ser663Leu), in the transactivation domain in two unrelated patients with severe obesity and strong family histories of the condition. Although in silico predictions for this variant are conflicting (deleterious according to SIFT/MutationTaster but benign according to PolyPhen-2), its absence in population databases (gnomAD, TOPMed) and co-segregation with obesity in affected siblings suggest a potential pathogenic role. Previous studies have demonstrated that *SIM1* missense variants can impair transcriptional activity, contributing to the development of obesity.

Interestingly, we identified a synonymous variant, c.1173G>A, p.(Ser391Ser), in five patients. Although synonymous variants are often presumed benign, in silico splicing prediction tools yield conflicting results regarding their potential effect on mRNA processing. For instance, Alamut Visual v2.11 predicted the creation of a cryptic acceptor site six base pairs downstream of the native splice site, suggesting possible aberrant splicing. However, other splice prediction algorithms, such as SpliceAI (low predicted probability of causing a major splice defect) and MaxEntScan (no strong signal for creating or destroying a splice site at this position), did not support a strong splice-altering effect. These discrepancies underscore the importance of further functional validation to determine whether this variant influences *SIM1* splicing in vivo. Similar observations of synonymous variants influencing splice sites have been reported previously, and these variants may influence splicing, protein function, and disease susceptibility [27,28,29,30]. These studies reinforce the notion that synonymous variants should not be ignored as neutral. Instead, integrative approaches that combine bioinformatics predictions, RNA-seq validation, and functional assays are essential for assessing their potential pathogenicity.

Our findings support previous evidence that *SIM1* disruptions lead to PWL phenotypes, including hyperphagia, early-onset obesity, and developmental delay [5,7,11]. The phenotypic overlap between *SIM1* and *MC4R* deficiency, another key component of the leptin-melanocortin pathway, further underscores the importance of *SIM1* in appetite regulation [31,32]. Notably, some of our patients exhibited endocrine abnormalities, including hypothyroidism and hypothalamic dysfunction, consistent with earlier reports of patients with *SIM1* deficiency [31,33].

To assess the structural impact of pathogenic *SIM1* missense variants, we analyzed both the monomeric and heterodimeric forms of SIM1. SIM1 forms heterodimers with ARNT or ARNT2 [34]. However, because ARNT2 is preferentially expressed in the hypothalamus and the variants under investigation alter hypothalamic function, we focused on its interaction with ARNT2. This dimerization is a prerequisite for DNA binding and transcriptional activity [13]. Since experimental structures of full-length SIM1 and ARNT2 are unavailable, we used AlphaFold3 to predict their domain structures. The AlphaFold3 models provide a structural perspective for interpreting the potential effects of *SIM1* missense variants in the context of its heterodimerization with ARNT2. Both proteins belong to the bHLH–PAS (basic helix–loop–helix/Per-Arnt-Sim) family of transcription factors and function as heterodimeric partners in neurodevelopment and hypothalamic regulation. They share typical domain architecture, comprising bHLH, PAS-A, PAS-B, and PAC [8,35]. The N-terminal region (first 59 residues) of ARNT2, along with the C-terminal regions of both SIM1 and ARNT2, are predicted to be intrinsically disordered.

Heterodimerization and DNA binding are mediated by the bHLH and PAS domains, while the PAC motif, which stabilizes the PAS domain fold, links the PAS tandem to the intrinsically disordered C-terminal transcriptional regulatory region [8,36]. To focus on the structured elements, we modeled the bHLH, PAS, and PAC domains of both proteins, excluding intrinsically disordered segments that do not contribute to heterodimerization and remain challenging to predict accurately in AlphaFold. This corresponded to residues 1–350 in SIM1 and 60–455 in ARNT2.

The identification of P30 at the dimer interface highlights a residue likely to play a stabilizing role in the bHLH-mediated heterodimer. Substituting this proline with alanine (P30A) could disrupt optimal packing at the interface, thereby weakening dimerization and diminishing subsequent transcriptional regulation. In fact, several studies have identified obesity-associated missense variants in the SIM1 bHLH (Thr46Arg, Glu62Lys) [8,36], and PAS (Ser71Arg, Ile128Thr, Gln152Glu, Arg171His, Leu238Arg) domains, all of which are involved in forming the heterodimer with ARNT2 [9].

In contrast, Ser663 lies within the intrinsically disordered C-terminal region of SIM1, which functions as a transcriptional regulatory region. Although AlphaFold predictions provide limited insight into disordered regions, previous functional studies have shown that C-terminal *SIM1* variants frequently disrupt transcriptional regulation rather than DNA binding or dimerization. For example, Arg550His, Pro692Leu, Asp707His, Gly715Val, and Asp740His have all been associated with obesity and varying effects on transcriptional activity [8,9,36,37,38]. These mutations cluster within the transactivation domain, underscoring the functional sensitivity of this region. It is therefore plausible that Ser663Leu, although structurally unresolvable, could similarly alter the recruitment of transcriptional cofactors or interfere with post-translational modifications required for SIM1-mediated gene regulation.

Overall, the modeling results indicate distinct mechanistic effects for the two variants analyzed. While the P30A mutation may compromise heterodimer formation, the Ser663Leu mutation likely affects transcriptional regulation by disrupting the C-terminal regulatory functions. These interpretations align with known pathogenic mechanisms of *SIM1* variants implicated in hypothalamic dysfunction and monogenic obesity. Further validation through co-immunoprecipitation, BRET/FRET interaction assays, and luciferase reporter experiments would be valuable for confirming the structural predictions and elucidating the specific molecular consequences of these variants.

### Limitations

Our study has limitations; functional assays are needed to confirm the pathogenicity of variants. Trio analysis was not available to investigate co-segregation of the variants. Such analyses would strengthen the evidence for causality, especially for variants with conflicting in silico predictions. The influence of polygenic background, epigenetic factors, or environmental influences on the obesity phenotype among carriers was not examined. These factors might contribute to the variable expressivity and penetrance of SIM1-related obesity.

## 5. Conclusions

This study broadens the range of *SIM1* variants linked to monogenic obesity, underscoring their role in hypothalamic development and energy regulation. Functional studies are needed to better understand how these variants, particularly the synonymous change, affect SIM1 function. Early genetic testing for *SIM1* mutations in children with severe obesity and hyperphagia could facilitate personalized interventions, leading to improved long-term metabolic outcomes.

## Figures and Tables

**Table 1:** *SIM1* Reported Variants Associated With Obesity.

Variants	Nucleotide Change	AA Change	rs	Phenotype	Reference
1	c.106C>T	p.Gln36*	NA	Early-onset Obesity	[17]
2	c.137C>G	p.Thr46Arg	rs1356756679	Obesity	[8]
3	c.184G>A	p.Glu62Lys	rs201038781	Obesity	[8]
4	c.213C>G	p.Ser71Arg	rs111927367	Obesity	[9]
5	c.214C>T	p.Pro72Ser	rs138378556	Early-onset obesity and hypopituitarism	[12]
6	c.280G>A	p.Val94Met	rs1189500818	Obesity	[18]
7	c.348+5G>A	N/A (splicing)	NA	Obesity	[19]
8	c.400G>A	p.Asp134Asn	rs1260456470	Early-onset obesity	[20]
9	c.454C>G	p.Gln152Glu	rs140908824	Obesity with Prader–Willi syndrome	[8]
10	c.490A>T	p.Met164Leu	rs533688069	Early-onset obesity	[21]
11	c.616C>A	p.Gln206Lys	rs757280640	Early-onset obesity	[19]
12	c.918G>T	p.Trp306Cys	NA	Early-onset obesity	[17]
13	c.967C>T	p.His323Tyr	rs756633599	Obesity	[8]
14	c.975_976insACTCCTG	p.(Val326Thrfs*43)	NA	Obesity	[22]
15	c.1027T>C	p.Ser343Pro	NA	Obesity	[22]
16	c.1054C>T	p.Pro352Ser	rs3734354	Prader–Willi-like syndrome	[23]
17	c.1121G>A	p.Arg374Gln	rs191644383	Obesity	[18]
18	c.1147A>G	p.Arg383Gly	rs188821440	Obesity	[9]
19	c.1442C>A	p.Thr481Lys	rs745899394	Early-onset obesity	[11]
20	c.1490C>G	p.Pro497Arg	NA	Obesity	[9]
21	c.1550C>T	p.Ala517Val	NA	Early-onset obesity	[11]
22	c.1622C>T	p.Ser541Leu	rs968598837	Obesity	[9]
23	c.1649G>A	p.Arg550His	rs137870558	Obesity	[9]
24	c.1846G>C	p.Gly616Arg	rs757303574	Early-onset obesity	[21]
25	c.2075C>T	p.Pro692Leu	rs757043702	Obesity	[9]
26	c.2108G>A	p.Arg703Gln	rs376058205	Obesity	[9]
27	c.2119G>C	p.Asp707His	rs74726213	Obesity	[9]
28	c.2135C>T	p.Thr712Ile	rs745821604	Obesity	[9]
329	c.2140A>G	p.Thr714Ala	rs757139012	Obesity with Prader–Willi syndrome	[9]
30	c.2144G>T	p.Gly715Val	rs753660670	Obesity	[18]
31	c.2218G>C	p.Asp740His	NA	Obesity	[8]

Note: NA = Not available.

**Table 2:** Conservation of the missense variants among several species.

**(A) c.88C>G, p. (Pro30Ala)**																									
**Species**	**Match**	**Amino Acid**																							
**Human**	**Wild Type**	**30**	**E**	**F**	**Y**	**E**	**L**	**A**	**K**	**L**	**L**	**P**	**L**	**P**	**S**	**A**	**I**	**T**	**S**	**Q**	**L**	D	K	A	S
mutated (p.P30A)	not conserved	30	E	F	Y	E	L	A	K	L	L	P	L	**A**	S	A	I	T	S	Q	L	D	K	A	S
Ptroglodytes	all identical	30	E	F	Y	E	L	A	K	L	L	P	L	**P**	S	A	I	T	S	Q	L	D	K	A	S
Mmulatta	all identical	30	E	F	Y	E	L	A	K	L	L	P	L	**P**	S	A	I	T	S	Q	L	D	K	A	S
Fcatus	all identical	30	E	F	Y	E	L	A	K	L	L	P	L	**P**	S	A	I	T	S	Q	L	D	K	A	S
Mmusculus	all identical	30	E	F	Y	E	L	A	K	L	L	P	L	**P**	S	A	I	T	S	Q	L	D	K	A	S
Ggallus	all identical	30	E	F	Y	E	L	A	K	L	L	P	L	**P**	S	A	I	T	S	Q	L	D	K	A	S
Drerio	all identical	30	E	F	Y	E	L	A	K	L	L	P	L	**P**	S	A	I	T	S	Q	L	D	K	A	S
Dmelanogaster	all identical	54	E	F	C	E	L	A	K	L	L	P	L	**P**	A	A	I	T	S	Q	L	D	K	A	S
Xtropicalis	all identical	30	E	F	Y	E	L	A	K	L	L	P	L	**P**	S	A	I	T	S	Q	L	D	K	A	S
**(B) c.1988C/T, p.(Ser633Leu)**																									
**Species**	**Match**	**Amino Acid**																							
**Human**	**Wild Type**	**663**	**P**	**T**	**A**	**L**	**S**	**R**	**I**	**S**	**S**	**P**	**N**	**S**	**D**	**R**	**I**	**S**	**K**	**S**	**S**	**L**	I	L	A
Mutated (p.S633L)	not conserved	663										P	N	**L**	D	R	I	S	K	S	S	L	I	L	A
Ptroglodytes	all identical	663										P	N	**S**	D	R	I	S	K	S	S	L	I	L	A
Mmulatta	all identical	663										P	N	**S**	D	R	I	S	K	S	S	L	I	L	A
Fcatus	all identical	635										P	N	**S**	D	R	I	S	K	S	S	L	I	L	A
Mmusculus	all identical	662										P	S	**S**	D	R	I	T	K	S	S	L	I	L	A
Ggallus	all identical	663									S	P	N	**S**	D	R	I	S	K	S	S	L	V	L	A
Xtropicalis	all identical	663								S	S	P	N	**S**	D	R	V	S	K	S	S	V	T	I	A

**Table 3:** Summary of Patient Demographics, Genetic Variants, and ACMG Classifications.

Cases	Age	Weight (Kg)	BMI (Kg/m^2^)	Variant	Coordinates (GRCh37)	SIFT	Polyphen2	MutationTaster	ACMG Variant Interpretation	gnomAD MAF
Case 1	13	112.8	35.7	c. 52_53delAG, p.Ser18Ter	6:100911292-100911293delCT	NA	NA	Disease Causing	PVS1 (Pathogenic)	0.0000
Case 2	15	159.0	47.3	c.88C>G, p.Pro30Ala	6:100911257-G-C	Deleterious	Probably Damaging	Disease Causing	PM1, PM2, PM3 (Likely pathogenic)	0.0000
Case 3	13	108.6	48.0	c.428_434del p.His143Ter	6:100897490-100897496delGGTTGAT	NA	NA	Disease causing	PVS1 (Pathogenic)	0.0000
Case 4 Case 5 Case 6	17 5 15	99.6 40.7 102	35.4 28.7 32.4	c.1988C>T p.Ser663Leu	6:100838550-G-A	Deleterious	Likely Benign	Disease Causing	PM2 (Uncertain Significance)	000003977
Case 7 Case 8 Case 9 Case 10 Case 11	11 10 15 17 14	71.7 65.0 136.0 103.0 106.0	32.0 31.5 48.8 37.0 35.0	c.1173G>A, p.Ser391Ser	6:100841760-C-T	NA	NA	Polymorphism	PM2, BP4, BP7 (Likely Benign)	0.000004305

NA: Not applicable.

## Data Availability

The original contributions presented in this study are included in the article. Further inquiries can be directed to the corresponding author.

## References

[1] World Health Organization 2021. https://www.who.int/news-room/fact-sheets/detail/obesity-and-overweight.

[2] Hinney A., Vogel C.I.G., Hebebrand J. (2010). From monogenic to polygenic obesity: Recent advances. Eur. Child Adolesc. Psychiatry.

[3] Oswal A., Yeo G.S.H. (2007). The leptin melanocortin pathway and the control of body weight: Lessons from human and murine genetics. Obes. Rev..

[4] Farooqi I.S., O’Rahilly S. (2005). Monogenic Obesity in Humans. Annu. Rev. Med..

[5] Faivre L., Cormier-Daire V., Lapierre J.M., Colleaux L., Jacquemont S., Geneviéve D., Saunier P., Munnich A., Turleau C., Romana S. (2002). Deletion of the *SIM1* gene (6q16.2) in a patient with a Prader-Willi-like phenotype. J. Med. Genet..

[6] Kim M.S., Rossi M., Abusnana S., Sunter D., Morgan D.G., Small C.J., Edwards C.M., Heath M.M., Stanley S.A., Seal L.J. (2000). Hypothalamic localization of the feeding effect of agouti-related peptide and alpha-melanocyte-stimulating hormone. Diabetes.

[7] Michaud J.L., Boucher F., Melnyk A., Gauthier F., Goshu E., Lévy E., Mitchell G.A., Himms-Hagen J., Fan C.M. (2001). *Sim1* haploinsufficiency causes hyperphagia, obesity and reduction of the paraventricular nucleus of the hypothalamus. Hum. Mol. Genet..

[8] Bonnefond A., Raimondo A., Stutzmann F., Ghoussaini M., Ramachandrappa S., Bersten D.C., Durand E., Vatin V., Balkau B., Lantieri O. (2013). Loss-of-function mutations in SIM1 contribute to obesity and Prader-Willi–like features. J. Clin. Investig..

[9] Ramachandrappa S., Raimondo A., Cali A.M., Keogh J.M., Henning E., Saeed S., Thompson A., Garg S., Bochukova E.G., Brage S. (2013). Rare variants in single-minded 1 (SIM1) are associated with severe obesity. J. Clin. Investig..

[10] Yang C., Gagnon D., Vachon P., Tremblay A., Levy E., Massie B., Michaud J.L. (2006). Adenoviral-Mediated Modulation of *Sim1* Expression in the Paraventricular Nucleus Affects Food Intake. J. Neurosci..

[11] Zegers D., Beckers S., Hendrickx R., Van Camp J.K., De Craemer V., Verrijken A., Van Hoorenbeeck K., Verhulst S.L., Rooman R.P., Desager K.N. (2014). Mutation screen of the SIM1 gene in pediatric patients with early-onset obesity. Int. J. Obes..

[12] Gonsalves R., Aleck K., Newbern D., Shaibi G., Kapadia C., Oatman O. (2020). Severe early onset obesity and hypopituitarism in a child with a novel SIM1 gene mutation. Endocrinol. Diabetes Metab. Case Rep..

[13] Michaud J.L., DeRossi C., May N.R., Holdener B.C., Fan C.M. (2000). ARNT2 acts as the dimerization partner of SIM1 for the development of the hypothalamus. Mech. Dev..

[14] Shimogori T., Lee D.A., Miranda-Angulo A., Yang Y., Wang H., Jiang L., Yoshida A.C., Kataoka A., Mashiko H., Avetisyan M. (2010). A genomic atlas of mouse hypothalamic development. Nat. Neurosci..

[15] Michaud J.L., Rosenquist T., May N.R., Fan C.M. (1998). Development of neuroendocrine lineages requires the bHLH–PAS transcription factor SIM1. Genes Dev..

[16] Tolson K.P., Gemelli T., Gautron L., Elmquist J.K., Zinn A.R., Kublaoui B.M. (2010). Postnatal *Sim1* Deficiency Causes Hyperphagic Obesity and Reduced *Mc4r* and Oxytocin Expression. J. Neurosci..

[17] Akıncı A., Türkkahraman D., Tekedereli İ., Özer L., Evren B., Şahin İ., Kalkan T., Çürek Y., Çamtosun E., Döğer E. (2019). Novel Mutations in Obesity-related Genes in Turkish Children with Non-syndromic Early Onset Severe Obesity: A Multicentre Study. J. Clin. Res. Pediatr. Endocrinol..

[18] Kleinendorst L., Massink M.P.G., Cooiman M., Savas M., Van Der Baan-Slootweg O.H., Roelants R.J., Janssen I.C.M., Meijers-Heijboer H.J., Knoers N.V.A.M., van Amstel H.K.P. (2018). Genetic obesity: Next-generation sequencing results of 1230 patients with obesity. J. Med. Genet..

[19] Serra-Juhé C., Martos-Moreno G.Á., Bou de Pieri F., Flores R., Chowen J.A., Pérez-Jurado L.A., Argente J. (2020). Heterozygous rare genetic variants in non-syndromic early-onset obesity. Int. J. Obes..

[20] Stanikova D., Buzga M., Krumpolec P., Skopkova M., Surova M., Ukropcova B., Ticha L., Petrasova M., Gabcova D., Huckova M. (2017). Genetic analysis of single-minded 1 gene in early-onset severely obese children and adolescents. PLoS ONE.

[21] Saeed S., Janjua Q.M., Haseeb A., Khanam R., Durand E., Vaillant E., Ning L., Badreddine A., Berberian L., Boissel M. (2022). Rare Variant Analysis of Obesity-Associated Genes in Young Adults with Severe Obesity from a Consanguineous Population of Pakistan. Diabetes.

[22] Foucan L., Larifla L., Durand E., Rambhojan C., Armand C., Michel C.T., Billy R., Dhennin V., De Graeve F., Rabearivelo I. (2018). High Prevalence of Rare Monogenic Forms of Obesity in Obese Guadeloupean Afro-Caribbean Children. J. Clin. Endocrinol. Metab..

[23] Geets E., Zegers D., Beckers S., Verrijken A., Massa G., Van Hoorenbeeck K., Verhulst S., Van Gaal L., Van Hul W. (2016). Copy number variation (CNV) analysis and mutation analysis of the 6q14.1–6q16.3 genes SIM1 and MRAP2 in Prader Willi like patients. Mol. Genet. Metab..

[24] Mohammed I., Haris B., Al-Barazenji T., Vasudeva D., Tomei S., Al Azwani I., Dauleh H., Shehzad S., Chirayath S., Mohamadsalih G. (2023). Understanding the Genetics of Early-Onset Obesity in a Cohort of Children from Qatar. J. Clin. Endocrinol. Metab..

[25] UniProt Consortium (2019). UniProt: A worldwide hub of protein knowledge. Nucleic Acids Res..

[26] Abramson J., Adler J., Dunger J., Evans R., Green T., Pritzel A., Ronneberger O., Willmore L., Ballard A.J., Bambrick J. (2024). Accurate structure prediction of biomolecular interactions with AlphaFold 3. Nature.

[27] Plotkin J.B., Kudla G. (2011). Synonymous but not the same: The causes and consequences of codon bias. Nat. Rev. Genet..

[28] Hunt R.C., Simhadri V.L., Iandoli M., Sauna Z.E., Kimchi-Sarfaty C. (2014). Exposing synonymous mutations. Trends Genet..

[29] Farooqi I.S., Keogh J.M., Yeo G.S.H., Lank E.J., Cheetham T., O’Rahilly S. (2003). Clinical Spectrum of Obesity and Mutations in the Melanocortin 4 Receptor Gene. N. Engl. J. Med..

[30] Sauna Z.E., Kimchi-Sarfaty C. (2011). Understanding the contribution of synonymous mutations to human disease. Nat. Rev. Genet..

[31] Farooqi S.I. (2015). Genetic, molecular and physiological mechanisms involved in human obesity: Society for Endocrinology Medal Lecture 2012. Clin. Endocrinol..

[32] Xi D., Gandhi N., Lai M., Kublaoui B.M. (2012). Ablation of Sim1 Neurons Causes Obesity through Hyperphagia and Reduced Energy Expenditure. PLoS ONE.

[33] El Khattabi L., Guimiot F., Pipiras E., Andrieux J., Baumann C., Bouquillon S., Delezoide A.L., Delobel B., Demurger F., Dessuant H. (2015). Incomplete penetrance and phenotypic variability of 6q16 deletions including SIM1. Eur. J. Hum. Genet..

[34] Hao N., Bhakti V.L.D., Peet D.J., Whitelaw M.L. (2013). Reciprocal regulation of the basic helix–loop–helix/Per–Arnt–Sim partner proteins, Arnt and Arnt2, during neuronal differentiation. Nucleic Acids Res..

[35] Turer E.E., San Miguel M., Wang K.W., McAlpine W., Ou F., Li X., Tang M., Zang Z., Wang J., Hayse B. (2018). A viable hypomorphic *Arnt2* mutation causes hyperphagic obesity, diabetes and hepatic steatosis. Dis. Model Mech..

[36] Coban M.A., Blackburn P.R., Whitelaw M.L., van Haelst M.M., Atwal P.S., Caulfield T.R. (2020). Structural Models for the Dynamic Effects of Loss-of-Function Variants in the Human SIM1 Protein Transcriptional Activation Domain. Biomolecules.

[37] Woods S.L., Whitelaw M.L. (2002). Differential Activities of Murine Single Minded 1 (SIM1) and SIM2 on a Hypoxic Response Element. J. Biol. Chem..

[38] Blackburn P.R., Sullivan A.E., Gerassimou A.G., Kleinendorst L., Bersten D.C., Cooiman M., Harris K.G., Wierenga K.J., Klee E.W., van Gerpen J.A. (2020). Functional Analysis of the SIM1 Variant p.G715V in 2 Patients with Obesity. J. Clin. Endocrinol. Metab..

